# Older Adults’ Perceptions of Falls and Falls Prevention: An Interview-Based Study

**DOI:** 10.1093/geroni/igaa057.961

**Published:** 2020-12-16

**Authors:** Danielle Catona

**Affiliations:** George Mason University, Laurel, Maryland, United States

## Abstract

The aim of this study was to gain an understanding of older Americans’ perceptions of falls and strength and balance exercise (SBE) as a means of falls prevention. Face-to-face, semi-structured interviews were conducted with 72 community-dwelling adults aged 65 to 89 years recruited from a variety of settings. Data were coded inductively to identify themes present within participants’ responses. This process included open coding and creating categories. Data revealed four themes related to falls: (1) others are at risk of falling, but not me, (2) people who fall experience bodily harm, (3) people who fall are a burden to others, and (4) people who fall end up in nursing homes. Four themes emerged related to benefits/facilitators of SBE: (1) SBE enables older adults to remain active and independent, (2) SBE provides an opportunity for older adults to socialize, (3) SBE has positive physical and mental health effects for older adults, and (4) healthcare providers advise older adults to perform SBE. There were three barriers associated with SBE: (1) having limited/no prior SBE experience, (2) having a pre-existing condition, and (3) disliking group-based, SBE classes. Study findings suggest older adults underestimate their risk of falling compared to their peers. As a result, SBE interventions may be promoted more effectively by highlighting personal and social benefits associated with SBE rather than physical risks associated with falls. Additionally, personal recommendations from healthcare providers as well as identification of modified and home-based programs may increase participation in SBE interventions.

